# The prion protein protease sensitivity, stability and seeding activity in variably protease sensitive prionopathy brain tissue suggests molecular overlaps with sporadic Creutzfeldt-Jakob disease

**DOI:** 10.1186/s40478-014-0152-4

**Published:** 2014-10-21

**Authors:** Alexander H Peden, Deep P Sarode, Carl R Mulholland, Marcelo A Barria, Diane L Ritchie, James W Ironside, Mark W Head

**Affiliations:** National CJD Research & Surveillance Unit (NCJDRSU), School of Clinical Sciences (Division of Clinical Neurosciences), College of Medicine and Veterinary Medicine, University of Edinburgh, Western General Hospital, Crewe Road, Edinburgh, EH4 2XU UK

**Keywords:** Variably protease resistant prionopathy, VPSPr, Prion, PrP, PrPSc, Gerstmann–Sträussler–Scheinker syndrome (GSS), PMCA, RT-QuIC

## Abstract

**Introduction:**

Variably protease sensitive prionopathy (VPSPr) is a recently described, sporadic human prion disease that is pathologically and biochemically distinct from the currently recognised sporadic Creutzfeldt-Jakob disease (sCJD) subtypes. The defining biochemical features of the abnormal form of the prion protein (PrP^Sc^) in VPSPr are increased sensitivity to proteolysis and the presence of an N- and C-terminally cleaved ~8 kDa protease resistant PrP^Sc^ (PrP^res^) fragment. The biochemical and neuropathological profile of VPSPr has been proposed to resemble either Gerstmann–Sträussler–Scheinker syndrome (GSS) or familial CJD with the *PRNP*-V180I mutation. However, in some cases of VPSPr two protease resistant bands have been observed in Western blots that co-migrate with those of type 2 PrP^res^, suggesting that a proportion of the PrP^Sc^ present in VPSPr has properties similar to those of sCJD.

**Results:**

Here, we have used conformation dependent immunoassay to confirm the presence of PrP^Sc^ in VPSPr that is more protease sensitive compared with sCJD. However, CDI also shows that a proportion of PrP^Sc^ in VPSPr resists PK digestion of its C-terminus, distinguishing it from GSS associated with ~8 kDa PrP^res^, and showing similarity to sCJD. Intensive investigation of a single VPSPr case with frozen tissue from multiple brain regions shows a broad, region-specific spectrum of protease sensitivity and differential stability of PrP^Sc^ in the absence of PK treatment. Finally, using protein misfolding cyclic amplification and real-time quaking induced conversion, we show that VPSPr PrP^Sc^ has the potential to seed conversion *in vitro* and that seeding activity is dispersed through a broad range of aggregate sizes. We further propose that seeding activity is associated with the ~19 and ~23 kDa PrP^res^ rather than the ~8 kDa fragment.

**Conclusions:**

Therefore, PrP^Sc^ in VPSPr is heterogeneous in terms of protease sensitivity and stability to denaturation with the chaotrope GdnHCl and includes a proportion with similar properties to that found in sCJD.

**Electronic supplementary material:**

The online version of this article (doi:10.1186/s40478-014-0152-4) contains supplementary material, which is available to authorized users.

## Introduction

Human prion diseases are invariably fatal neurodegenerative disorders that include sporadic, genetic and acquired forms. The phenotypic and strain-related properties of human prion diseases are, according to the prion hypothesis, enciphered in the conformation of the misfolded, disease associated isoform of the prion protein, PrP^Sc^ (Table 1), which is partially protease resistant and accumulates in the brains of patients with disorders such as Creutzfeldt-Jakob disease and Gerstmann-Sträussler-Scheinker syndrome (GSS). Molecular strain typing has focused extensively on differences in the fragment size and glycosylation site occupancy of PrP^res^, the proteinase resistant core of PrP^Sc^ (Table 1) resulting from limited digestion with proteinase K (PK). Although defined by clinico-pathological criteria, different subtypes of human prion disease can be classified using these PrP^res^ molecular profiles in conjunction with the presence of mutations and polymorphisms in the prion protein gene (*PRNP*). For example, in sporadic CJD (sCJD) six molecular subtypes have been defined that combine the *PRNP* codon-129 genotype polymorphism (MM, MV or VV) with the apparent molecular mass of the unglycosylated protease resistant fragment of PrP^res^ on western blots which is either 21 kDa (type 1) or 19 kDa (type 2A), according to the nomenclature of Parchi and Gambetti [1]. In addition, other PrP^Sc^ fragment sizes have been noted in association with other human prion diseases, e.g. GSS with the P102L mutation in *PRNP*, in which either type 1 PrP^res^ or a low molecular mass ~8 kDa PrP^res^ fragment can predominate [2].

**Table 1:** **Abbreviations used throughout**

Abbreviation	Definition
PrP^Sc^	The abnormal, disease-associated form (generically termed the scrapie isoform, irrespective of the disease and species in which it occurs) which is characterised by increased β-pleated sheet content, decreased solubility and increased protease-resistance compared with PrP^C^ as a result of refolding of the protein and self-aggregation.
PrP^C^	Normal form of the prion protein as expressed in the central nervous system and other tissues.
PrP^res^	Protease resistant core of PrP^Sc^ detected by western blotting following treatment with 50 μg/ml PK.
~8 kDa PrP^res^ fragment	Low molecular mass PrP^res^ fragment of ~8 kDa detected by western blotting following treatment with 50 μg/ml PK in brain tissue from patients with VPSPr and some patients with GSS.
senPrP^Sc^	The component of PrP^Sc^ that is poorly resistant to protease treatment (i.e. >2.5 μg/ml PK)
CDI	Conformation dependent immunoassay
GSS	Gerstmann-Sträussler-Scheinker syndrome
GSS (~8 kDa PrP^res^)	GSS case associated with a low molecular mass fragment of PrP^res^ of approximately ~8 kDa when analysed by western blotting following proteinase K digestion.
GSS (type 1 PrP^res^)	GSS case characterised by the presence of 20-30 kDa type 1 PrP^res^ fragments in brain when analysed by western blotting following proteinase K digestion.

Variably protease-sensitive prionopathy (VPSPr) is a novel sporadic human prion disease that was first reported in the USA in 2008 [3]. Further cases of VPSPr have been identified prospectively and retrospectively in the USA [4], UK [5]-[7], the Netherlands [8] and Spain [9],[10]. VPSPr has a distinctive neuropathology characterised by the presence of microplaques, but it is essentially defined by the biochemistry of PrP^Sc^ in the brain that is less resistant to proteases than the PrP^Sc^ in other human prion diseases, and the presence of N- and C-terminally cleaved ~8 kDa PrP^res^. In these respects VPSPr has been proposed to resemble GSS [4],[8], although a comparison has also been drawn with familial CJD associated with the V180I-129 M haplotype, with respect to the absence of diglycosylated PrP^res^ and the presence of ~8 kDa PrP^res^[11]. Alternatively, we and others have identified regional heterogeneity in the PrP^res^ profile in some VPSPr brains, specifically the presence of bands with mobilities very similar to type 2A PrP^res^ in VPSPr cerebellum, an observation that suggests that a proportion of the PrP^Sc^ present has properties similar to those found in sCJD [3],[7],[10].

In this paper, we have employed conformation dependent immunoassay (CDI), to characterise the physicochemical properties of PrP^Sc^ in VPSPr brain. CDI can sensitively detect both protease-sensitive PrP^Sc^ (senPrP^Sc^) and protease resistant PrP^Sc^ (PrP^res^) with intact C-termini, and can measure the conformational stability of PrP^Sc^ isoforms to guanidine denaturation when no PK is used. CDI detects PrP^Sc^ on the basis of an increase in signal following denaturation with guanidine HCl, because epitopes hidden within the structure of PrP^Sc^ become exposed in the presence of this chaotropic salt. In practice, for CDI to efficiently detect PrP^Sc^ a preparative step, such as NaPTA precipitation, mild proteolysis (≤2.5 μg/ml PK) or centrifugal concentration is usually necessary, in order to remove PrP^C^[12]-[14]. We have performed the above analyses comparing VPSPr to sCJD and GSS focussing on aspects of the regional variability of PrP^Sc^ in terms of protease resistance and stability. These analyses have been done in order to aid our understanding of the distinction of VPSPr from other prion diseases at the molecular level. We have also investigated whether there are any regional differences in the way VPSPr PrP^Sc^ behaves in a cell-free conversion assay, protein misfolding cyclic amplification (PMCA). Having previously shown that brain homogenates of all sCJD subtypes can efficiently seed real-time quaking induced conversion (RT-QuIC) at levels equivalent to femto grams (1x10^-15^ g) of PrP^res^, we also compared the seeding potential of differently sized PrP^Sc^ aggregates in VPSPr and sCJD by RT-QuIC [15].

## Materials and methods

### Tissues used in this study

This study was limited by the availability of frozen tissue with consent for research from VPSPr cases. We have identified nine cases of VPSPr in total over the surveillance period 1991-present, disproportionately affecting individuals who are VV at *PRNP* codon-129. Table 2 summarises the frozen tissue available for this study. No MM cases and only one MV case had frozen tissue available for research. In only one of the four VV cases (case 1) was a complete half brain taken at autopsy with consent for research.

**Table 2:** **Summary of the five VPSPr cases used in this study**

VPSPr case number (in this study)	***PRNP***-codon 129 genotype	References	Frozen tissues available (Abbreviations as in Figure5.)	Semi-quantitative score for the presence of microplaques in the cerebellum 0 = absent 3+ = severe
1	VV	This study	FC, TC, PC, OC, Cb, Hip,Thal, Midbrain, Pons, Me	1+
2	VV	[7] (Case 4)	FC, Cb	3+
3	VV	[7] (Case 3)	FC, Cb, TC	1+
4	VV	[5]	FC, Cb	3+
5	MV	[6]	FC	0

In addition, three sporadic Creutzfeldt–Jakob disease (sCJD) cases (MM1, MM2 and VV2 subtypes), one variant CJD case (vCJD), two Gerstmann–Straüssler–Scheinker disease (GSS) cases (both P102L mutation), and 10 control (non-prion disease) cases were analysed in this study. Five of the latter control cases, from the MRC Edinburgh CJD Brain and Tissue bank, had been considered for a clinical diagnosis of human prion disease, but an alternative pathological diagnosis was reached. The other five cases, from the MRC Sudden Death Brain and Tissue bank, had no neurological or neuropathological evidence of disease. All cases used were of UK origin. The tissues were collected with consent for research, and the study was conducted under research ethics approval (11/ES/0022, Edinburgh Brain Bank).

### Immunohistochemistry

VPSPr cases in this study were reviewed by immunohistochemical analysis for PrP using the anti-PrP antibodies 3F4, 12F10, KG9 and 6H4 as described previously [7]. A semi-quantitative estimation was made on the relative density of microplaques within the molecular layer of the cerebellum in all five cases of VPSPr using the 3F4 antibody; sections were reviewed independently by two experienced reviewers (DLR, JWI) using a four point scale with 0 being absent and 3+ being severe (see Table 2).

### Homogenization of brain samples for conformation dependent immunoassay (CDI) analysis

Frozen tissue samples were weighed and homogenised in phosphate buffered saline containing 2% *N*-lauroylsarcosine, to a final tissue concentration of 10% (wt/vol) using a Fastprep machine (MP Biomedical, CA, USA) as described by us previously [12]. The homogenates were then cleared of cellular debris by centrifugation at 5200 *g* for five minutes at 4°C.

### Detection of PrPSc by CDI

We used a 96-well plate based conformation-dependent immunoassay (CDI) to characterise the physicochemical properties of PrP^Sc^ in VPSPr and the controls mentioned above. The CDI method used has been described previously [13]. CDI resembles a sandwich ELISA but it involves a capture antibody, MAR-1, which binds both the native and denatured forms of the normal prion protein, PrP^c^ (Table 1), and the abnormal, disease-associated prion protein, PrP^Sc^. However, the detection antibody (europium-labelled 3F4) binds both native and denatured PrP^c^, but only binds to PrP^Sc^ after it has been denatured by guanidine hydrochloride (GdnHCl). Therefore, the signal detected when the sample is denatured (D) minus the signal for the native samples (N) can be used as a quantitative measure of PrP^Sc^.

**Figure 1:**
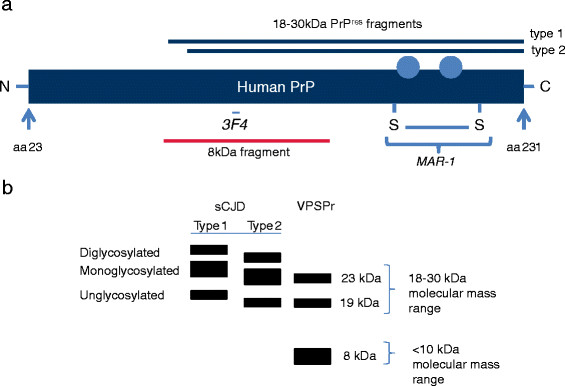
**Human PrP primary structure. (a)** Schematic diagram of human PrP primary structure showing the location of epitopes for the MAR-1 capture antibody (used in CDI) and the 3F4 detection antibody (used in CDI and western blotting) and the regions corresponding to the 18-30 kDa PrP^res^ bands and the ~8 kDa PrP^res^ band. Blue circles indicate the position of the glycan moieties at amino acids 181 and 197. Note that MAR-1 epitope is discontinuous and depends on an intact C-terminal disulphide bond, which is absent from the ~8 kDa PrP^res^ fragment seen in cases of VPSPr. Due to the position of the MAR-1 capture epitope, only PrP^Sc^ with an intact C-terminus is detectable by CDI. **(b)** Schematic diagram indicating the approximate migration positions of bands obtained when samples from sCJD brain or VPSPr brain are treated with PK and analysed by immunoblotting using the anti-PrP monoclonal antibody 3F4 (epitope amino acids 106-112). The upper ~19 and ~23 kDa bands observed in some VPSPr cases have mobilities similar the unglycosylated and monoglycosylated bands of type 2A PrP^res^ in sCJD.

For PrP^Sc^ to be detected by CDI, the MAR-1 and 3F4 epitopes must be intact and not subject to proteolytic processing in either conformer. Due to the position of the MAR-1 capture epitope, only PrP^Sc^ with an intact C-terminus is detectable by CDI (Figure 1a). The ~8 kDa fragment observed in VPSPr by western blot following proteinase K digestion (Figure 1b) lacks that C-terminal epitope for the MAR-1 antibody and is undetectable by CDI. However, the bands observed in some brain regions from some VPSPr cases at ~19 and ~23 kDa which may directly correspond to the mono- and unglycosylated bands of sCJD with type 2 PrP^res^ (Figure 1b) are detectable using antibodies that recognise C-terminal epitopes in PrP (Additional file 1: Figure S1). Therefore, these protease resistant fragments may be detectable by CDI.

Homogenates (10% w/v) from all tissues used in this study (see above) were either assayed by CDI directly, or following limited proteolytic digestion with 1, 2.5, 10 or 50 μg/ml PK for 1 h at 37°C. PK digestion was terminated by the addition of 1 mM Pefabloc SC. Homogenates were divided into two equal aliquots. One aliquot was mixed with an equal volume of 8 M guanidine hydrochloride (GdnHCl) and denatured (D) by incubating at 81°C for six minutes and the other was diluted without GdnHCl and left at room temperature and designated native (N). The remainder of the CDI method used in this study was performed as described previously [13] using the dissociation enhanced lanthanide fluorescence immunoassay technology (DELFIA™, PerkinElmer, Cambridge, UK). Both D and N samples were adjusted to a final volume of 650 μl and a final guanidine concentration of 308 mM, with water containing complete™ EDTA-free protease inhibitors (Roche, Hertfordshire, UK). The anti-PrP capture antibody MAR-1 (a generous gift from Dr Albrecht Gröner, CSL Behring, Marburg, Germany) was used at 0.5 μg/well. The wells were washed with DELFIA wash buffer (PerkinElmer). After saturating the plate by shaking for 1 h with 0.5% (wt/vol) bovine serum albumin and 6% D-sorbitol (wt/vol) in DELFIA wash buffer, the wells were washed and the D or N samples were loaded in triplicate onto the plate (200 μl/well) and incubated for 2 h at 20°C with shaking.

After washing the plate, europium-conjugated anti-PrP antibody 3F4 (~25 ng/ml) in DELFIA assay buffer (200 μl/well) was added to all wells and incubated for 2 h at 20°C with shaking. The wells were then washed six times and DELFIA enhancement solution (200 μl/well) was added to all wells. After incubation for five minutes at 20°C with shaking, the time-resolved fluorescence signals for the denatured and native samples were measured using a Victor 2 fluorometer (PerkinElmer).

The value obtained from the denatured sample (D) represents the detection of total PrP i.e. PrP^C^ + PrP^Sc^. The value obtained for the native sample (N), without denaturation, represents PrP^C^. Therefore, D-N is directly proportional to PrP^Sc^. In reality, there may be a small increase in the accessibility of the 3F4 epitope in unfolded PrP^C^ versus native PrP^C^. Therefore, to be effective, a preparative step is necessary to remove the majority of PrP^C^ prior to analysis.

### Calibration of CDI

The CDI assay for PrP was calibrated using full-length human recombinant PrP (recHuPrP, amino acids 23-231, methionine at codon 129) purified as described previously [15]. A range of dilutions from 40 μg/ml to 10 μg/ml of this recHuPrP was prepared using PBS containing 2% sarkosyl. Twenty-five μl samples of these dilutions were mixed with an equal volume of 8 M GdnHCl and denatured as described above. These samples were adjusted to 650 μl, with water containing complete™ EDTA-free protease inhibitors and analysed as described above. The time resolved fluorescence counts obtained for the recHuPrP dilution series were used to plot a standard curve of counts versus micrograms of PrP. This curve was used to convert the ‘D’ and ‘N’ CDI signals obtained for known volumes of 10% (wt/vol) brain homogenates into values with units of micrograms of PrP per gram of brain tissue.

### Equilibrium unfolding for the analysis of PrPSc stability

Stability analysis of the PrP^Sc^ conformer was performed on two cases of GSS with the P102L mutation (one associated with an ~8 kDa PrP^res^ and the other associated with the typical three-band type 1 PrP^res^ following western blot analysis) and on various brain regions from VPSPr case 1, for which multiple brain regions were available (Table 2). Frontal cortex and cerebellum from a case of sCJD (VV2 subtype) and two cases of GSS were analysed as controls. To concentrate PrP^Sc^, the brain tissue homogenates (100 μl) were centrifuged (1 h at 20,000 *g* at 4°C) [16]. The pellets were resuspended in 50 μl of 0.1% N-lauroylsarcosine/PBS containing complete™ EDTA-free protease inhibitors and a range of GdnHCl concentrations from 0 to 4 M or 6 M and incubated with shaking (~500 rpm) overnight at 20°C. The samples were adjusted to a final volume of 650 μl and then loaded onto MAR-1 coated plates and analysed by DELFIA™ using Eu-3F4 as described above. The data were normalised to ‘fraction of PrP^Sc^ unfolded’ by adjusting the value obtained without denaturation (0 M GdnHCl) to zero and the maximum value to 1. The data were analysed by non-linear regression assuming a sigmoidal relationship between GdnHCl concentration and the fraction of PrP^Sc^ unfolded.

### Western blotting

The western blot method that was used to analyse the multiple brain regions from VPSPr case 1 has been described previously [17]. The protocol used was one specifically designed to detect poorly protease-resistant PrP that occurs in the form of a ~8 kDa band in cases with variably protease-sensitive prionopathy. For western blot analysis, samples were homogenized to 10% (w/v) in tris-buffered saline, pH 7.6, containing 0.5% Nonidet P40 and 0.5% sodium deoxycholate and cleared by centrifugation at 400 *g* for 5 min. Aliquots of the cleared 10% brain homogenates were subjected to limited proteolysis with PK at 50 μg/ml for 1 h at 37°C terminated with 1 mM Pefabloc SC (Roche, Burgess Hill, UK). Unless otherwise stated, maximal volumes of 24 μl were loaded onto the gel. Polyacrylamide gel electrophoresis and Western blotting was performed using the NuPAGE Novex gel system (Life Technologies, Paisley, UK). The gel electrophoresis time was shortened to retain low molecular mass proteins [5],[8]. The immunodetection of PrP was achieved using the monoclonal antibodies 3F4 (epitope 106-112, from Millipore, Watford, UK) at final concentrations of 75 ng/ml or 94B4 (epitope 187-194, from Central Veterinary Institute of Wageningen UR, Lelystad, The Netherlands) at a concentration of 1:5000 for 1 h. The secondary antibody used was ECL Mouse IgG, HRP-linked whole Ab (GE Healthcare Life Sciences, Amersham, United Kingdom). The detection reagent employed was Amersham ECL Prime western Blotting Detection Reagent (GE Healthcare Life Sciences). The blots were imaged and analysed by densitometry using the ChemiDoc™ XRS + System with Image Lab™ Software (Bio-Rad, Hemel Hempstead, UK).

### Protein misfolding cyclic amplification (PMCA)

PMCA was carried out as described previously [18]. VPSPr cases 2 and 3, both VV at *PRNP*-codon 129, were selected in which a distinction had been observed in the western blot PrP^res^ pattern between the frontal cortex (predominantly ~8 kDa fragment) and the cerebellum (bands of 18-30 kDa, in a pattern similar to type 2A). The two VPSPr cases, numbered 2 and 3 in this study, correspond to cases 4 and 3, respectively, in the retrospective review of UK cases (Table 2) [7]. Brain homogenate was prepared from the frontal cortex (FC) and cerebellum (Cb) of VPSPr cases 2 and 3 for use as seeds. For comparison, PMCA was also carried out on frontal cortex tissue from a case of sCJD (VV2 subtype) and two cases of GSS with the P102L mutation, one associated with ~8 kDa and the other associated with type 1 PrP^res^. The brain homogenates used as substrates were prepared from either frontal cortex of non-CJD patients, or humanised transgenic mouse brain, with compatible *PRNP*-codon 129 genotypes (VV in the case of VPSPr and sCJD VV2 subtype) and MM for both cases of GSS). The (seed:substrate) volume ratios were as follows: VPSPr cases (1:4), GSS and sCJD cases (1:9). Low molecular mass heparin (100 μg/ml) was included in all PMCA reactions [19].

### Real-time quaking induced conversion (RT-QuIC)

We used RT-QuIC to examine the seeding activity of VPSPr case 1, versus a sCJD VV2 case and a non-CJD case (amyotrophic lateral sclerosis with frontotemporal lobar dementia). The method used for the RT-QuIC *in vitro* conversion assay was as described previously [15] with some modifications. Full length hamster recombinant PrP (aa 23–231; GenBank accession no. K02234) was exclusively used as a substrate. This was expressed and purified as described previously [15], and provided by Dr Gary Mallinson, NHS Blood and Transplant, Bristol, UK. Brain seeds were prepared by homogenising frozen tissue samples using a Fastprep machine (as described above) in PBS containing 1 mM EDTA, 150 mM NaCl, 0.5% Triton X-100 and Complete Mini EDTA-free Protease Inhibitor Cocktail (Roche) to give a final tissue concentration of 10% (w/v). The amounts of brain homogenate introduced into the RT-QuIC assays were normalized according to the concentration of PrP^res^ determined by quantitative analysis of western blots in which brain samples had been run against known amounts of recombinant PrP [15]. Dilutions of the brain homogenates were performed using PBS containing 0.1% sodium dodecyl sulphate (SDS). The RT-QuIC reactions were a final volume of 100 μl and contained recombinant PrP at a final concentration of 0.1 mg/ml. The reactions were initiated by the addition of 2 μl of the appropriately diluted seed. The plates were incubated at 42°C and shaken intermittently at 900 r.p.m. (87 sec shaking, 33 min at rest) using a FLUOstar OMEGA microplate reader (BMG Labtech) in a double orbital configuration. Fluorescence readings were taken at 480 nm every 15 min from the bottom of the wells after excitation with 20 flashes per well at 450 nm. Thioflavin-T emission counts (relative fluorescence units) increased to a maximum limit of 260,000 per well.

### Sucrose density gradient centrifugation (SDGC)

SDGC was performed as described previously [12] to separate PrP^Sc^ aggregates according to their densities from samples of frontal cortex from VPSPr case 1, and case of sCJD VV2. Frontal cortex from a non-CJD case was separated as a control. Briefly, centrifugation was carried out in an Optima™ TLX Ultracentrifuge (Beckman Coulter) using an MLS-50 swinging bucket rotor. The sucrose gradient used was set up in a 5 ml ultracentrifuge tube. Each step was 745 μl and the dilutions were, from bottom to top, 60, 30, 25, 20, 15 and 10% sucrose in Tris-buffered saline containing 1% N-lauroylsarcosine. Whole brain homogenate 5% (w/v) containing 2% N-octyl glucopyranoside (NOG) in PBS (1X) was clarified and loaded on top of the sucrose gradient and centrifuged at 4°C and 50,000 g for 1 hr, all as described in [12]. During centrifugation the sucrose gradient becomes continuous. After centrifugation 11 fractions of 450 μl were collected by pipetting from the top of the gradient. Therefore, the density of the fractions increases from 1-11. For western blot analysis, fractions were PK digested (50 μg/ml PK, 1 h) and precipitated with nine volumes of methanol overnight at -80°C and pellets were collected by centrifugation (16000 g for 1 hr). For RT-QuIC analysis, fraction samples were diluted 1:10 with PBS containing 0.1% SDS.

## Results

### D-N values for VPSPr frontal cortex compared with other prion diseases

**Figure 2:**
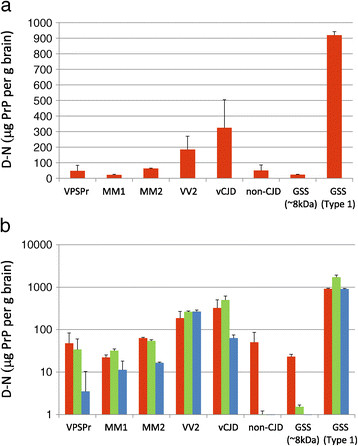
**Comparison of the D-N values in brain frontal cortex from cases of VPSPr, sCJD MM1, sCJD MM2, sCJD VV2, vCJD, two GSS cases and non-CJD. (a)** The D-N values for VPSPr patients are the means (+/- S.D.) of five patients, whereas the values for non-CJD are from ten patients, five being cases of sudden death, three Alzheimer’s disease, one cerebral infarction and one motor neurone disease/frontal lobe dementia. Also shown for comparison, are D-N values for individual patients with vCJD (MM2B), sCJD subtypes MM1 and MM2, and VV2, and two cases of GSS with the P102L mutation of *PRNP,* one GSS (~8 kDa) and the other GSS (type 1). For the latter results from individual patients, the D-N values are the means (+/- S.D.) of triplicate analyses of single samples. **(b)** The D-N values (log_10_ scale) are shown for the same brain frontal cortex samples as in **(a)**, but additionally D-N values obtained after treating the homogenates with no PK (red bars) are compared with the values obtained after 2.5 or 50 μg/ml PK (green and blue bars), respectively.

Figure 2 shows the D-N values of VPSPr frontal cortex brain homogenates (mean of samples from five patients). These values are compared with frontal cortex from patients with sCJD (of the MM1, MM2 and VV2 subtypes), variant CJD, and two cases of GSS (both P102L mutation of *PRNP*). Cases with a diagnosis other than prion disease (non-CJD) are included as a negative control. This analysis was performed after no treatment (Figure 2a), or treatment with 2.5 or 50 μg/ml PK (Figure 2b). In the absence of PK digestion, CDI is ineffective at discriminating between prion disease and non-prion disease specimens: using the mean D-N value for non-CJD cases plus 3 s.d. as a cut-off value (158 μg PrP per gram brain) only sCJD VV2 subtype, vCJD and GSS (type 1 PrP^res^) give values above this cut-off.

In contrast, CDI could clearly distinguish between prion disease and non-prion disease specimens after pretreament of the homogenates with 2.5 μg/ml PK (Figure 2b, green bars). After this mild PK treatment, the D-N signals were abolished for non-CJD samples, but high D-N values were obtained for VPSPr equivalent to those obtained for sCJD MM1 and sCJD MM2. Using the mean [D-N] (+3 s.d.) for non-CJD samples pretreated with 2.5 μg/ml PK as the new cut-off , samples from all prion diseases apart from GSS (~8 kDa) gave values above this threshold. The high D-N signals from the VPSPr, CJD and GSS specimens following denaturation of samples digested with 2.5 μg/ml PK indicated the presence of PrP^Sc^ that resists mild proteolysis, in contrast to PrP^c^ which is abolished. Therefore, low concentration PK treatment allows D-N to distinguish between the prion disease samples (VPSPr, CJD, GSS) and the non-CJD group.

High concentration PK treatment (50 μg/ml) abolishes D-N in the GSS case associated with ~8 kDa PrP^res^ (Figure 2b, blue bars). However, between 2.5 and 50 μg/ml PK, D-N is maintained in sCJD VV2 subtype and GSS (type 1 PrP^res^), but falls in all other prion diseases, the fall being greatest for VPSPr. We can infer that the decrease in D-N between 2.5 and 50 μg/ml PK is due to the elimination of poorly protease-resistant forms of PrP^Sc^, referred to as senPrP^Sc^ (Table 1). Alternatively, rather than eliminating PrP^Sc^, 50 μg/ml PK digestion may remove the C-terminal epitope for the capture antibody, rendering PrP^Sc^ undetectable by CDI. This would be the case if the digestion product was the ~8 kDa fragment observed on western blots for VPSPr and GSS. It is interesting to note that after 50 μg/ml PK, very high D-N signals were detected in the GSS (type 1 PrP^res^) case, in contrast with the GSS (~8 kDa PrP^res^) case, in which D-N was completely abolished at 50 μg/ml PK (Figure 2).

### D-N values for frontal cortex versus cerebellum in VPSPr cases

**Figure 3:**
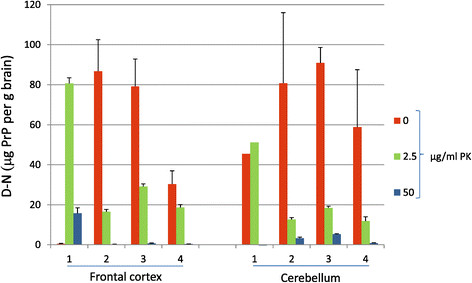
**Comparison of VPSPr frontal cortex and cerebellum by CDI.** CDI analysis of samples of frontal cortex or cerebellar cortex homogenate from VPSPr cases 1-4 following treatment with 0 (red bars), 2.5 (green bars) or 50 μg/ml PK (blue bars) for 1 h. The error bars are S.D. for triplicate analyses of single samples.

**Figure 4:**
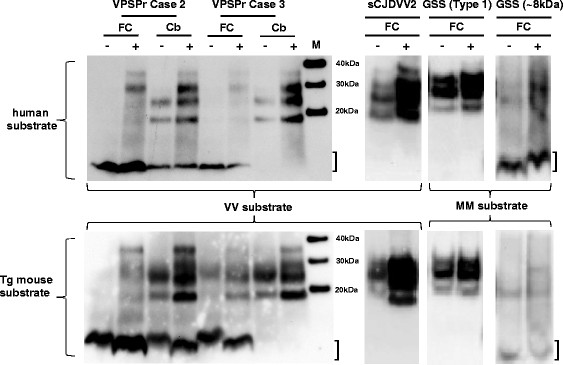
**PMCA of VPSPr, GSS and sCJD PrP** ^**Sc**^ **.** Brain homogenate prepared from the frontal cortex (FC) and cerebellum (Cb) of VPSPr cases 2 and 3, and frontal cortex from two cases of GSS (associated with ~8 kDa and type 1 PrP^res^, respectively) and a case of sCJD VV2 were used to seed single-round PMCA reactions. The brain homogenates used as substrates are as indicated in the figure, and were either non-CJD human post mortem brain frontal cortex tissue (upper panel) or humanised transgenic mouse brain expressing human *PRNP* (lower panel). Substrates with compatible *PRNP*-codon 129 genotypes were used (VV in the case of VPSPr and sCJD VV2, and MM for both cases of GSS). The (seed:substrate) volume ratios were as follows: VPSPr cases (1:4), GSS and sCJD cases (1:9). Samples were amplified by PMCA (+) or kept frozen (-). The region of the ~8 kDa PrP^res^ band is indicated with a bracket. Separate immunoblots have been aligned to the molecular mass markers shown in lane M.

**Figure 5:**
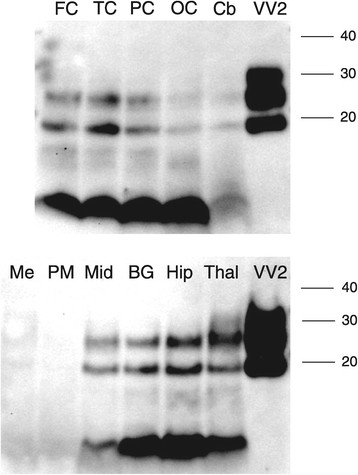
**Western blot analysis.** The brain regions analysed were cerebral cortex (frontal cortex, FC, temporal cortex, TC, parietal cortex, PC, occipital cortex, OC), cerebellum, (Cb), hippocampus (Hip), thalamus (Thal), midbrain, pons major (PM), and medulla (Me). Samples were homogenised in extraction buffer, digested with 50 μg/ml PK, and separated by gel electrophoresis and immunoblotted using 3F4 as the primary antibody. Frontal cortex from a case of sCJD VV2 was analysed in the lane on the far right for both western blots (VV2). The positions of the molecular mass markers (in kDa) are indicated on the right.

CDI analysis comparing frontal cortex and cerebellum for VPSPr cases 1-4, following digestion with 0, 2.5 and 50 μg/ml PK is shown in Figure 3. Similar to frontal cortex, positive D-N value are obtained for cerebellum following treatment with 2.5 μg/ml PK (Figure 3, red bars). In the frontal cortex of VPSPr cases 2-4 and in the cerebellum of VPSPr case 1 and 4, the D-N values are severely reduced following treatment with 50 μg/ml PK. For VPSPr cases 2 and 3 the D-N values were considerably greater for cerebellum versus frontal cortex after 50 μg/ml PK (Figure 3) suggesting the presence of protease resistant PrP^Sc^ with intact C-termini. In these cases, a marked distinction had been seen in the western blot PrP^res^ molecular profile for the frontal cortex (~8 kDa PrP^res^) and cerebellum (predominantly triple band pattern resembling type 2A PrP^res^) (Figure 4) [7]. However, in VPSPr case 1, the western blot profile showed the presence of ~19 and ~23 kDa bands in addition to the ~8 kDa band in the frontal cortex (Figure 5), and in agreement with this, CDI analysis showed the preservation of the D-N signal in frontal cortex after treatment with 50 μg/ml PK.

### PMCA of VPSPr PrPSc from frontal cortex and cerebellum

VPSPr cases 2 and 3 described above were used to investigate the relative potential for PrP^Sc^ from the frontal cortex or the cerebellum to seed the conversion of PrP^C^ in a PMCA reaction. These cases were selected for this test because of the extreme differences between frontal cortex and cerebellum seen in the western blot PrP^res^ pattern and the D-N values after treatment with 50 μg/ml PK (Figure 3). No increase in the abundance of the ~8 kDa PrP^res^ in the frontal cortex was observed after PMCA using either non-CJD human brain or humanised transgenic brain substrate (Figure 4). However, the ~19 and ~23 kDa bands in the VPSPr cerebellum that co-migrate with middle and lower bands of the type 2A in sCJD (Figure 1B) were amplified by PMCA using both substrates. Amplification was apparent as the appearance of a band at ~30 kDa and an increase in signal for the ~19 and ~23 kDa bands. The mean increase in signal (VPSPr cases 2 and 3) for the latter two bands was ~2.6- and 1.5-fold, using human and mouse brain substrates, respectively. A ~30 kDa band, that may correspond to diglycosylated PrP^res^, also appears when VPSPr frontal cortex samples are amplified by PMCA (Figure 4). The replicative properties of PrP^Sc^ from cases of GSS associated with either type 1 or ~8 kDa PrP^res^ were also investigated by PMCA. Densitometric analysis indicated a ~1.5 fold level of amplification, using either human or mouse brain substrate for GSS with type 1 PrP^res^ in contrast to a lack of amplification for GSS with an ~8 kDa PrP^res^, using human and transgenic mouse brain substrates (Figure 4). In contrast, both the human brain and humanised transgenic mouse brain substrates showed robust amplification when seeded with a sCJD VV2 subtype sample (Figure 4).

### Neuropathological correlate

An interesting question is whether the biochemical and seeding potential differences observed between VPSPr frontal cortex and cerebellum are reflected in regional neuropathology. We were unable to see a clear correlation between the detection of cerebellar ~19 and 23 kDa PrP^res^ by western blot, (high is cases 2 and 3) and the number of cerebellar microplaques between cases (high in cases 2 and 4) (Table 2).

### Regional variation in a VPSPr case with extensive available tissue

#### Western blot analysis

Unlike the other VPSPr cases identified by surveillance in the UK, multiple brain regions were available for analysis from VPSPr case 1 (Table 2). Eleven anatomical regions from the brain of case 1 were analysed. Samples were homogenised and prepared for conventional western blot analysis. PrP^res^ was widely distributed throughout cortical and subcortical regions with regional variation in the relative levels of the ~8 kDa PrP^res^ and two bands that appeared to co-migrate with the unglycosylated and mono-glycosylated PrP^res^ bands of the sCJD VV2 subtype at ~23 and ~19 kDa (Figure 5). The band corresponding to diglycosylated PrP was not visible. The brain regions with the highest overall signal intensities were hippocampus > occipital cortex > parietal cortex, whereas PrP^res^ was largely absent from the medulla and pons. The ~8 kDa band was predominant in most regions, but its predominance was highest for the occipital cortex and lowest for the midbrain and thalamus (Figure 5).

### Comparison of D-N values from multiple regions from a single case of VPSPr

**Figure 6:**
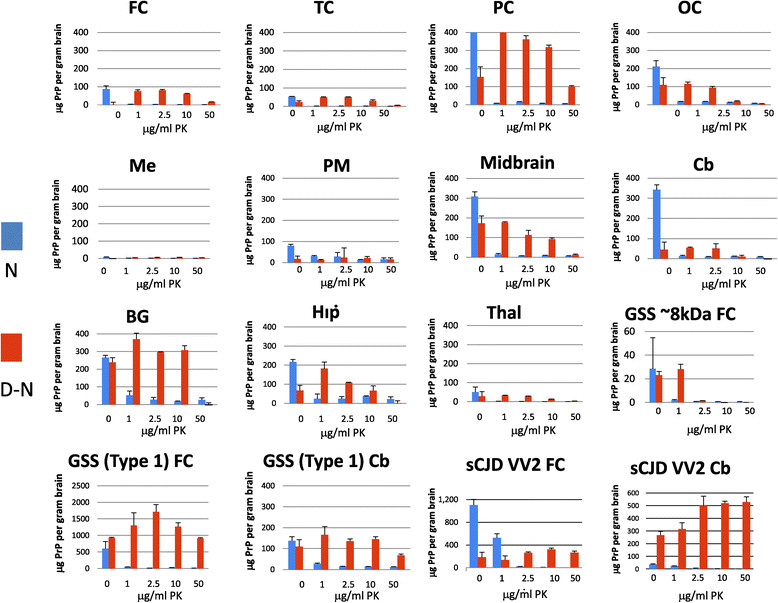
**CDI analysis of brain regions from a VPSPr case 1 after treatment of the homogenates with various concentrations of PK.** The brain regions analysed were cerebral cortex (frontal cortex, FC, temporal cortex, TC, parietal cortex, PC, occipital cortex, OC), cerebellum, (Cb), hippocampus (Hip), thalamus (Thal), midbrain, pons major (PM), and medulla (Me). Homogenates were prepared in 2% N-lauroylsarcosine/PBS and treated with 0, 1, 2.5, 10 or 50 μg/ml PK for 1 h at 37°C. In addition, samples of frontal cortex were analysed from the GSS (~8 kDa PrP^res^) case, and samples of frontal cortex and cerebellum were analysed from the GSS (type 1 PrP^res^) case and a case of sCJD VV2 subtype. The results are the mean D-N values (+/- S.D.) for triplicate analyses of single samples.

Samples from VPSPr case 1 were taken from exactly the same anatomical regions as those analysed by western blotting and analysed by CDI, for both PrP^Sc^ stability and relative levels of PrP^Sc^, as inferred from D-N. These samples were compared with available samples from the two GSS cases used above. Samples were homogenised and underwent digestion with 0, 1, 2.5, 10 or 50 μg/ml PK. PrP^Sc^ was detectable in all VPSPr brain regions apart from medulla using CDI analysis (Figure 6), which is in partial agreement with the western blot analysis for PrP^res^. The highest D-N values were detected in the parietal cortex and basal ganglia, and also the occipital cortex and midbrain. The lowest D-N levels were detected in the thalamus and medulla. Negligible D-N signals were obtained for frontal cortex from a case of amyotrophic lateral sclerosis with frontotemporal lobar dementia (ALS-FTLD) used as a control (data not shown).

To be detected by CDI the protease resistant PrP^Sc^ must contain the epitope to 3F4 and the C-terminal epitope to MAR-1 making the ~8 kDa PrP^res^ fragment, characteristic of VPSPr and GSS cases, undetectable by CDI (Figure 1a). In GSS (type 1 PrP^res^) and sCJD VV2 there is CDI detectable material even at the highest 50 μg/ml PK concentration. However, in GSS (~8 kDa PrP^res^) little or none survives 2.5 μg/ml PK. Regional analysis of VPSPr case 1 shows them to be intermediate in profile: after treatment with 50 μg/ml PK, PrP^Sc^ remained detectable in the parietal cortex in a manner that resembles GSS (type 1 PrP^res^) and sCJD VV2 subtype (Figure 6), and after 10 μg/ml PK, PrP^Sc^ was detected in midbrain, basal ganglia and hippocampus. However, a high PK concentration largely eliminated CDI-detectable PrP^Sc^ from the other regions, and this PK sensitivity therefore resembles GSS (~8 kDa PrP^res^).

### Comparison of PrPSc stability in multiple regions from a single case of VPSPr

PrP^Sc^ is insoluble in detergents such as *N*-lauroylsarcosine and can be concentrated by centrifugation. We confirm this here by showing that PrP^Sc^ (inferred from positive D-N values) is pelleted from VPSPr frontal cortex homogenate, but is absent from the centrifugal pellet obtained from non-CJD brain homogenate (Additional file 1: Figure S2).

**Figure 7:**
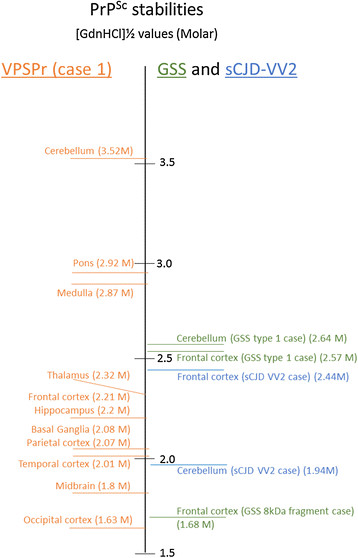
**Comparison of PrP** ^**Sc**^ **stabilities to GdnHCl denaturation in various brain regions from a VPSPr and GSS cases.** The [GdnHCl]½ values obtained from various brain regions from a VPSPr case, two GSS cases (type 1 and ~8 kDa PrP^res^ case) and a sCJD VV2 case are plotted to scale (units of molar concentration of GdnHCl) on a vertical axis from [GdnHCl]½ =1.5 M at the bottom to [GdnHCl]½ =3.5 M. The [GdnHCl]½ values for VPSPr are shown in red on the left, whereas the GSS and sCJD VV2 values are on the right in green and blue, respectively. [GdnHCl]½ values (along with 95% confidence intervals for the curve fit) are also listed in Table 3.

The insoluble pellet can then be used to measure the stability of VPSPr PrP^Sc^ in the absence of PK. Samples were centrifugally concentrated from 100 μl aliquots of homogenate, prior to denaturation with increasing concentrations of GdnHCl. By increasing the signal for PrP^Sc^ and removing detergent soluble PrP, centrifugal concentration enabled the determination of PrP^Sc^ stability in regions such as medulla in which PrP^Sc^ had been close to, or below, the limit of detection in the previous CDI analysis (Figure 6). The equilibrium denaturation curves for the analysis of brain regions from VPSPr case 1 are shown in Additional file 1: Figure S3 along with the [GdnHCl]½ values and the 95% confidence intervals (CI). The [GdnHCl]½ values and CI values are also summarised in Table 3 and Figure 7. Although PrP^Sc^ stability analysis in VPSPr was restricted to a single case (case 1) where multiple regions were available, extensive analysis of another human prion disease subtype, vCJD, gives an indication of the precision of this method. For example, analysis of vCJD frontal cortex gave a mean [GdnHCl]½ value of 1.678 (S.D., ±0.175) for three separate vCJD cases [12]. All regions of this VPSPr case (and GSS and sCJD positive controls) show a simple sigmoidal transition when unfolded by increasing guanidine concentrations. The regional [GdnHCl]½ values for this VPSPr case varied dramatically, being lowest in occipital cortex ([GdnHCl]½ =1.63) and highest in the cerebellum ([GdnHCl]½ =3.52) (Table 3). Analysis of multiple adjacent samples from frontal cortex and cerebellum from VPSPr case 1 showed that the difference in the [GdnHCl]½ values for these regions was statistically significant (Table 4). In the GSS (type 1 PrP^res^) case, no distinction was seen between the stabilities of frontal cortex and cerebellum PrP^Sc^, although in both cases it was high compared with PrP^Sc^ in the GSS ~8 kDa PrP^res^ frontal cortex (Table 3). In sCJD VV2 subtype frontal cortex, PrP^Sc^ was more stable than cerebellar PrP^Sc^, the latter being in the same range as VPSPr frontal cortex (Table 3). This suggests that the difference observed in VPSPr case 1 is not an effect of cerebellum *per se*, but of the actual stability of the PrP^Sc^ that is present in different neuroanatomical regions of this VPSPr brain.

**Table 3:** **Summary of [GdnHCl]½ values for brain regions from the VPSPr case and the GSS cases**

Brain region	[GdnHCL]½ value	95% confidence interval for the curve fit.
VPSPr frontal cortex	2.21	0.11
VPSPr temporal cortex	2.01	0.42
VPSPr parietal cortex	2.07	0.39
VPSPr occipital cortex	1.63	0.21
VPSPr Me	2.87	0.35
VPSPr pons	2.92	0.23
VPSPr Midbrain	1.8	0.61
VPSPr cerebellum	3.52	0.11
VPSPr basal ganglia	2.08	0.21
VPSPr Hip	2.2	0.26
VPSPr Thal	2.32	0.31
sCJD VV2 FC	2.44	0.22
sCJD VV2 Cb	1.94	0.1
GSS frontal cortex (type 1 PrP^res^ case)	2.57	0.06
GSS cerebellum (type 1 PrP^res^ case)	2.64	0.06
GSS frontal cortex (~8 kDa frag. case)	1.68	0.36

The [GdnHCl]½ values shown here were obtained from the curve fits shown in Additional file 1: Figure S3. The data used to obtain the curve fits were from single samples, or in the case of VPSPr FC, Cb and PC, data merged from the analyses of 4, 3 or 2 samples, respectively.

**Table 4:** **PrP** ^**Sc**^ **stability analysis of multiple samples from frontal cortex and cerebellum from VPSPr case 1**

	FC (n = 4)	Cb (n = 3)	T-Test (FC vs Cb)
[GdnHCl]½ ± S.E.	2.28 ± 0.08	3.60 ± 0.14	*P* =0.003

The data shown are the mean [GdnHCl]½ values (± standard error) for separate curve fits obtained from separate samples of frontal cortex (FC) and cerebellum (Cb) from VPSPr case 1.

### Samples from VPSPr contain seeding activity in RT-QuIC

**Figure 8:**
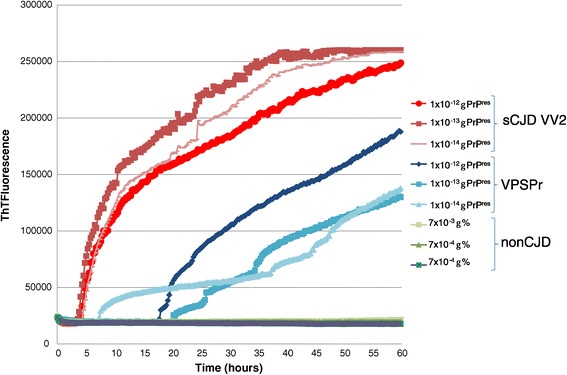
**VPSPr PrP** ^**Sc**^ **can seed conversion in RT-QuIC.** The reactions were seeded with three 10-fold dilutions of VPSPr or sCJD VV2 brain amount, normalised according the titre of PrP^res^ (units shown are grams PrP^res^ per 100 μl reaction volume). As a control, equivalent dilutions of non-CJD brain homogenate (shown as% [w/v]) were used to seed parallel reactions. Mean ThT fluorescence values (relative fluorescence units) for the quadruplicate reactions were plotted against time (hours).

We investigated whether VPSPr could also seed conversion of recombinant PrP in RT-QuIC. After normalising for the concentration of PrP^res^ (as determined by quantitative western blot), dilutions of either VPSPr case 1 or sCJD VV2 brain frontal cortex homogenate were used to seed the RT-QuIC reactions. Both VPSPr and sCJD VV2 brain homogenates seeded conversion. The efficiency of seeding on the basis of the lag time to a rise in ThT fluorescence over 60 hours, was lower for VPSPr per unit PrP^res^ compared with sCJD VV2 (Figure 8). No conversion was observed for reactions seeded with equivalent dilutions of non-CJD brain homogenate or unseeded reactions.

### RT-QuIC seeding activity of VPSPr and VV2 sCJD after sucrose density gradient separation (SDGC)

**Figure 9:**
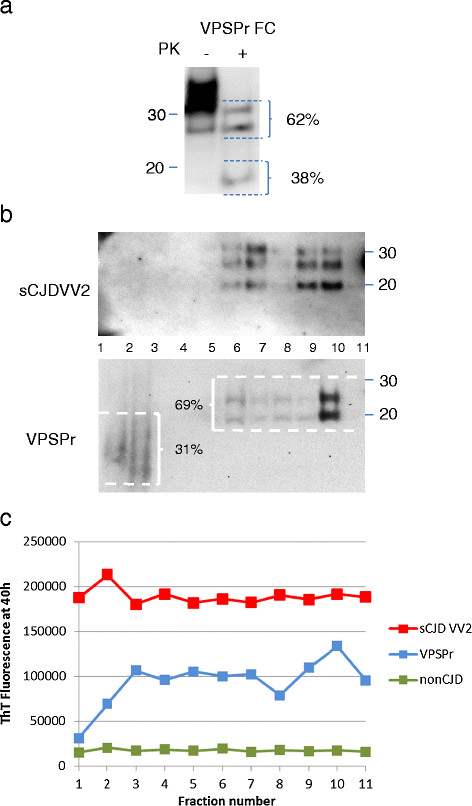
**RT-QuIC seeding activity of VPSPr and VV2 sCJD after sucrose density gradient separation. (a)** An adjacent sample from VPSPr frontal cortex to the one analysed below [in (b)] was homogenised to 10% w/v in extraction buffer and analysed by immunoblotting using 3F4 as the primary anti-PrP monoclonal antibody either without prior PK digestion (15 μl) or following digestion with 50 μg/ml PK and centrifugal concentration (from 100 μl). The densitometric analysis of the ~19 and ~23 kDa bands versus the ~8 kDa band in this sample is superimposed on the figure. **(b)** Samples of SDGC fractions from VPSPr case 1 (200 μl) and sCJD VV2 (100 μl) were PK treated (50 μg/ml, 1 h) precipitated overnight with nine volumes of methanol, and analysed for PrP^res^ by western blotting. The positions of molecular mass markers (in kDa) are indicated on the right. The collective densitometric analysis of the ~19 and ~23 kDa bands versus the ~8 kDa bands in the SDGC-separated sample is superimposed on the figure. **(c)** VPSPr (case 1), sCJD VV2 and non-CJD brain homogenate were separated by sucrose density gradient centrifugation (SDGC). The gradient used was 10-60% sucrose and after centrifugation eleven fractions of increasing density were successively taken from the top of the tube (#1 = lowest density, #11 highest density). Duplicate RT-QuIC reactions were seeded with 2 μl samples of 1:10 dilutions of the fractions. The ThT relative fluorescence values plotted are the means of the duplicate reactions at 40 hours.

SDGC was used to separate PrP^Sc^ aggregates from VPSPr and sCJD VV2 subtype brain according to the relative densities of aggregates. Figure 9a shows the direct western blot analysis of homogenate from a sample of frontal cortex from VPSPr case 1 that was adjacent to the sample used in the SDGC analysis. Western blot analysis of the SDGC fractions indicated that sCJD VV2 subtype brain PrP^Sc^ is predominantly in lower (heavier or more dense) fractions as judged by PK digestion and PrP^res^ detection by western blot (Figure 9b, upper panel). However, VPSPr PrP^Sc^ separates into a ~19 and ~23 kDa PrP^res^ form found in the lower fractions and an ~8 kDa PrP^res^ form in the upper (lighter or less dense) fractions as judged by PK digestion and western blot (Figure 9b, lower panel). No detectable PrP^Sc^ was found in the bottom (most dense) fraction. Densitometry of the ~8 kDa and 19/23 kDa PrP^res^ components before (38%:62%) and after (31%:69%) centrifugation suggests that the balance of these two distinct PrP^res^ subpopulations is largely unaffected by SDGC.

The fractions obtained following SDGC separation of samples of VPSPr, sCJD VV2 and non-CJD brain homogenate were investigated for their seeding potential, using the RT-QuIC assay. Using ThT fluorescence at 40 hours as a measure, seeding potential was associated with a broad range of densities for both the sCJD VV2 subtype and VPSPr (Figure 9b). In the case of sCJD VV2, all fractions seeded RT-QuIC. For VPSPr case 1 seeding activity was markedly reduced in the uppermost fractions (1 and 2) in which the ~8 kDa PrP^res^ was found.

## Discussion

### Biochemical and functional aspects of the PrPSc types present in VPSPr

These results firstly show that VPSPr PrP^Sc^ is detectable by CDI, but only after mild treatment with proteinase K. Therefore VPSPr, in common with all other human prion diseases, is associated with a misfolded form of PrP in which the 3F4 epitope (amino acids 106-112) is concealed. After treatment with 2.5 μg/ml PK, the D-N values for VPSPr were comparable to those for sCJD, MM1 and MM2, but less than those obtained for sCJD VV2 subtype, vCJD and GSS (type 1 PrP^res^).

It is interesting to note that in the case of sCJD VV2 and GSS (type 1 PrP^res^) high D-N signals remained regardless of the concentration of PK used. The particularly high values obtained for the sCJD VV2 subtype are in agreement Saverioni *et al* who showed that VV2 has the highest PK-resistance among sCJD types and that VPSPr is much less resistant [20]. However, in our study we have shown that in a case of GSS (with ~8 kDa PrP^res^) any CDI detectable PrP^res^ was eliminated with even mild PK treatment. Therefore, VPSPr appears to be intermediate between these two extremes: Although a considerable proportion of VPSPr PrP^Sc^ was sensitive to proteolysis with high PK, some PrP^Sc^ remained.

In two VPSPr cases, the PrP^Sc^ detectable after high PK treatment was considerably greater for cerebellum than frontal cortex. Western blot analysis of these cases had previously shown that frontal cortex PrP^res^ was predominantly ~8 kDa, whereas cerebellum PrP^res^ had a triple band pattern consistent with type 2A PrP^res^[7]. Interestingly, in VPSPr case 1, bands at ~19 and ~23 kDa, co-migrating with the lower and middle bands of type 2A PrP^res^ were observed in frontal cortex by western blotting. These bands were detectable using an antibody directed against the C-terminus of PrP. CDI analysis of frontal cortex from VPSPr case 1 following digestion with 50 μg/ml PK indicated the presence of resistant PrP^Sc^ with an intact C-terminus. Therefore, CDI in combination with stringent PK digestion is a sensitive method for mapping PrP^res^ in VPSPr that has a protease-resistant C-terminus, and in that respect resembles that found in sCJD.

### In vitro seeding activity and aggregate size

We are the first to show that brain homogenate from VPSPr can seed the conversion of PrP^C^ to PrP^Sc^ in a PMCA reaction. When the above mentioned two VPSPr cases, with extreme differences between frontal cortex and cerebellum PrP^res^ patterns, were used to seed PMCA reactions, only the ~19 and ~23 kDa bands present in the cerebellum were amplified with the additional appearance of a band at ~30 kDa. Our results indicate cerebellum and frontal cortex PrP^Sc^ have different seeding activities, with the amplification products of cerebellum resembling the triple-band pattern of CJD. These results mirrored the results obtained for GSS (~8 kDa PrP^res^) versus GSS (type 1 PrP^res^), in which only the latter seeded conversion in PMCA to a low but measurable extent. The different PMCA results obtained for the two anatomical brain regions in VPSPr and the subtypes of GSS suggests that different PrP^Sc^ isoforms may give rise to the ~8 kDa band and the type 1 or 2A PrP^res^ profiles observed by Western blotting, and that these PrP^Sc^ isoforms may have different replicative properties. An interesting question is whether the biochemical and seeding potential differences observed between VPSPr frontal cortex and cerebellum reflect neuropathological observations. We were unable to demonstrate a correlation between the detection of ~19 and ~23 kDa PrP^res^ and the presence of cerebellar microplaques between cases. Although our results suggest the co-existence of different PrP^Sc^ conformers in separate anatomical regions of VPSPr brains, we are unable to state whether they correspond to separate prion strains. To answer this, it would be necessary to demonstrate the transmission of different agent strains to experimental animals inoculated with samples from separate regions of VPSPr brains.

Both VPSPr and a form of familial CJD associated with the V180I-129 M haplotype (fCJD-V180I), shared a similar PrP^res^ electrophoretic profile on western blots with a low molecular mass band at ~8 kDa [10] and both lacked diglycosylated PrP and the N181 monoglycoform. Xiao *et al.* have suggested that VPSPr and fCJD-V180I share a pathway for the propagation of PrP^Sc^ in which the conversion of diglycosylated PrP^C^ and one of the two possible forms of monoglycosylated PrP^C^ (mono 181) to PrP^res^ is inhibited, which explains the absence or under-representation of a band co-migrating with diglycosylated PrP^res^ in the VPSPr electrophoretic profile [11]. The appearance of a strong diglycosylated band in the amplified product suggests that in PMCA this inhibition effect is overcome and that VPSPr PrP^Sc^ is capable of recruiting and converting diglycosylated and N181 monoglycosylated PrP^C^ to a highly protease-resistant form resembling that found in CJD.

We have also shown for the first time that VPSPr brain homogenate has the potential to seed conversion of recombinant PrP in the RT-QuIC assay. In the single cases that we examined a lower overall efficiency of conversion was observed for VPSPr compared with sCJD of the VV2 subtype. The analysis of a greater number of cases would be necessary to draw definite conclusions on the relative seeding potential of VPSPr versus other human prion disease subtypes. However, different conversion efficiencies between different human prion disease subtypes have been observed previously in RT-QuIC, with variant CJD converting very much less efficiently compared with sCJD [15].

For both VPSPr and sCJD VV2 the RT-QuIC seeding activity was widely dispersed throughout a 10-60% sucrose gradient, following ultracentrifigation and did not simply reflect the apparent sedimentation of PrP^res^ to the lower (more dense) fractions. The sedimentation properties of PrP^Sc^ from this VPSPr case 1 showed some similarities to those of sCJD VV2: For sCJD VV2, type 2A PrP^res^ was predominantly found in the lower (heavier or more dense) fractions. For VPSPr, bands at ~19 kDa and 23 kDa, that appeared to co-migrate with the lower and middle bands of type 2A PrP^res^ in sCJD, were also predominantly found in the lower fractions. However, the ~8 kDa PrP^res^ appeared to have different sedimentation properties and was confined to the upper (less dense) fractions. This implies that these two aggregation states (one which is recognised as ~8 kDa PrP^res^ and the associated with PK –resistant bands at ~19 kDa and ~23 kDa) differ in size and exist independently *in situ*. However, we cannot exclude the possibility that these two different forms might be disaggregated by the mild solubilising effects of the detergent (NOG) needed for SDGC.

The presence of seeding activity in the upper fractions suggests that less dense (presumably smaller) forms of protease sensitive PrP^Sc^, possibly including oligomers, can provoke PrP^C^ to PrP^Sc^ conversion and possibly contribute to the pathogenesis in human prion diseases. It is interesting to note that VPSPr seeding activity was reduced in the uppermost two fractions characterised by ~8 kDa PrP^res.^ The fact that ~8 kDa PrP^res^ also failed of amplify by PMCA may suggest that the PrP^Sc^ conformer associated with ~8 kDa PrP^res^ may be an “off pathway” end product of protein misfolding, and perhaps not, as one might suppose an intermediate between fully protease sensitive PrP^Sc^ and fully protease resistant PrP^Sc^.

VPSPr was first reported in 2008 and to date there are no published studies of its transmissibility to humanised transgenic mouse models. This may be taken to imply that VPSPr is at best poorly transmissible, specifically in comparison to CJD. The data reported here using *in vitro* seeding assays (PMCA and RT-QuIC) seem to suggest that VPSPr transmissibility might be associated with a biochemical profile in VPSPr that partly resembles that found in CJD.

### Regional variation of PrPSc abundance and type

The western blot analysis of regions from VPSPr case 1 showed PrP^res^ to be widespread throughout the brain, with the highest levels in the parietal cortex, and the lowest in the medulla and pons. The regional variations of the upper ~19 kDa and ~23 kDa PrP^res^ bands and the ~8 kDa PrP^res^ band suggest that these subtypes display independent variability according the location within the brain. The degree of difference in the molecular profile of PrP^res^ from frontal cortex and cerebellum does however differ between VPSPr cases. This difference was not a major feature of VPSPr case 1, the only case for which we had access to a full range of neuroanatomical regions.

Our study is the first to show a truly widespread distribution of PrP^res^ in a VPSPr brain by western blotting. In the original description of VPSPr cases, western blot analysis had been carried out on three subcortical regions (substantia nigra, putamen and thalamus) in eight cases of VPSPr and had found readily detectable amounts of PrP^res^ in only one of these subcortical regions (thalamus) from one case [3]. In another case report, PrP^res^ was detected by western blotting as a faint ladder-like pattern of bands in cerebral cortex and thalamus and as a PrP^res^ pattern resembling the triple band pattern of type 2A in cerebellum [9]. Based on the positions of the two immunoreactive bands (~19 and ~23 kDa) that are apparent in the VPSPr cerebellum of cases 2 and 3, and all anatomical regions analysed from VPSPr case 1, it is reasonable to conclude that there are molecular overlaps between VPSPr and sCJD: These bands appear to co-migrate with the middle and lower type 2A PrP^res^ bands (Figure 5 and ref [7]) and have intact C-termini (Additional file 1: Figure S1). However, it should be noted that, in contrast with sCJD VV2, there is a clear under-representation of the upper band.

The western blot results for VPSPr case 1, were reflected in the CDI analysis of the same multiple brain regions from this case. PrP^Sc^ (determined on the basis of D-N after mild protease treatment) was detected by CDI in the cerebral cortex, midbrain, hippocampus and basal ganglia, but was absent in the medulla. In this VPSPr case, there was a marked reduction, or elimination, of CDI-detectable PrP^Sc^ from many regions following treatment with the highest concentration of PK (50 μg/ml), whereas in the parietal cortex PrP^Sc^ remained. These results are consistent with the presence of two conformer classes in this case of VPSPr that have either C-terminal PK resistance (type 2 PrP^res^) or C-terminal PK sensitivity (~8 kDa PrP^res^).

The relative conformational stabilities of PrP^Sc^ from different regions from VPSPr case 1 were also determined by centrifugal concentration of PrP^Sc^, and the resuspension and denaturation of the pellet with a range of GdnHCl concentrations. We have shown that in the absence of PK, CDI-detectable PrP^Sc^ can be recovered from VPSPr homogenates by virtue of sarkosyl insolubility and following resuspension and unfolding in various concentrations of GdnHCl a sigmoidal transition is seen for all VPSPr regions and the GSS and sCJD VV2 subtype positive controls. Differential PrP^Sc^ stability was marked in VPSPr case 1, consistent with different forms or mixtures of forms present in different regions.

A marked difference in conformational PrP^Sc^ stability was seen between VPSPr cerebral cortex and cerebellum with cerebellar PrP^Sc^ being apparently much more stable and a [GdnHCl]½ of ~3.6 M. In contrast, in the sCJD VV2 subtype case we have shown that a marginally lower stability ([GdnHCl]½ ~1.9) is obtained for cerebellum compared with frontal cortex (~2.4) (Table 3 and Additional file 1: Figure S3). No difference in stability was observed between the frontal cortex and cerebellum for cases of GSS (type 1 PrP^res^) and previous studies failed to detect a marked difference in PrP^Sc^ stability between these regions in GSS [21] and variant CJD [12].

The co-existence of distinct PrP^res^ types (1 and 2A) has been noted in a significant proportion of sporadic CJD brains by western blotting [22],[23]. Using an alternative method to CDI, known as conformational stability immunoassay (CSI) Cali *et al* have reported different stabilities for type 1 versus type 2A PrP^res^ in individuals who are MM at *PRNP*-codon-129. In MM cases with co-existent PrP^res^ types a spectrum of PrP^Sc^ stabilities were obtained with values that spanned the region between the stabilities reported for (non-mixed) type 1 and type 2A PrP^res^ sCJD cases [22]. However, our study is the first to report a marked difference in PrP^Sc^ stability between two anatomical regions of the same prion disease brain and provides further evidence for the existence of PrP^Sc^ heterogeneity in VPSPr brains.

### The relationship of VPSPr to GSS

A similarity had been noted between GSS and VPSPr, in terms of the PrP^res^ electrophoretic profile and the relative sensitivities of PrP^res^ fragments within these profiles to digestion with PK [4],[24]. This has led to suggestions that VPSPr is the sporadic equivalent of GSS, in the same way that sporadic fatal insomnia (sCJD MM2-Thalamic) has been proposed to be the sporadic equivalent of fatal familial insomnia (*PRNP* D178N Codon-129M). Pirisinu *et al.* examined the relationship between VPSPr and GSS in humans and an atypical form of scrapie in sheep and goats known as Nor98 [21]. They also compared the conformation stabilities of PrP^Sc^ from a single brain region (frontal cortex) of VPSPr and GSS patients, using an alternative method to CDI, known as conformational stability and solubility assay (CSSA) that measures the diminution of PrP^Sc^ remaining in the detergent insoluble pellet after treatment with increasing concentrations of GdnHCl. In agreement with our study, Pirisinu *et al* determined the PrP^Sc^ stability in VPSPr frontal cortex (*PRNP*-codon 129 VV) to be [GdnHCl]½ =2.0-2.4 M [21] which they showed to be midway between the PrP^Sc^ stabilities in GSS P102L (type 1 + ~8 kDa PrP^res^) and GSS P102L (~8 kDa PrP^res^).

Our current study and the study by Pirisinu *et al.* indicate that the biochemical characteristics of PrP^Sc^ from the VPSPr cases did not exactly match the PrP^Sc^ phenotypes observed in GSS, and therefore suggest that VPSPr is a distinct biochemical entity. A key distinction we observed in this study is the differing conformational stabilities of VPSPr PrP^Sc^ between cerebral cortex and cerebellum and no such difference in PrP^Sc^ stabilities was observed for a case of GSS P102L (type 1 PrP^res^) (Table 3). PrP^Sc^ from VPSPr cerebellum is considerably more stable, and in a number of VPSPr cases it is associated with PrP^res^ bands on western blot analysis, that appear to co-migrate with type 2 PrP^res^ bands observed in sCJD. In contrast, PrP^Sc^ from VPSPr cerebral cortex has a stability and PrP^res^ molecular profile that is closer to GSS ~8 kDa PrP^res^ cases.

## Conclusions

Our analyses indicate the following: Firstly, CDI confirms the presence of readily detectable PrP^Sc^ in VPSPr that can be diminished by mild protease treatment. However, CDI also demonstrates that a proportion of the PrP^Sc^ found in VPSPr resists digestion of its C-terminus even when high concentrations of protease are used. This characteristic distinguishes VPSPr from GSS associated with ~8 kDa PrP^res^ and points to biochemical similarities with sCJD. Some, but not all cases of VPSPr show in the cerebellum PrP^res^ bands at ~19 and ~23 kDa that co-migrate with the lower and middle bands of type 2A PrP^res^ in sCJD, instead of, or in addition to, the ~8 kDa type. It is the former type that appears to replicate in PMCA. In contrast, little or no amplification was observed for the ~8 kDa PrP^res^ either in VPSPr or a case of GSS associated with this PrP^res^ subtype.

Intensive investigation of a single VPSPr case showed broad brain region-specific spectrum of protease sensitivity, differing relative amounts of ~8 kDa PrP^res^ and ~19 & 23 kDa PrP^res^, and the stability of PrP^Sc^ in the absence of proteases depending on the brain region examined. PrP^Sc^ was found to be widely distributed throughout cortical and some subcortical brain regions in VPSPr. The sedimentation properties and RT-QuIC seeding activity of PrP^Sc^ from VPSPr frontal cortex resemble those of CJD.

The overall conclusion of this study is that VPSPr is heterogeneous in terms of protease sensitivity and resistance to denaturation by chaotropes and includes a proportion of PrP^Sc^ with biochemical properties and functional characteristics similar to those of sCJD. This heterogeneity exists both between VPSPr cases and between brain regions within individual cases. Although this study has focussed on the biochemical aspects of PrP^Sc^ in VPSPr, the heterogeneity that we have observed may party underlie the neuropathological heterogeneity in VPSPr cases.

## Authors’ contributions

AHP, planned and executed the experiments working with the other authors, and drafted the manuscript; DPS, performed CDI analysis of both PrP^Sc^ sensitivity and stability and helped draft the manuscript, CRM performed the SDGC and RT-QuIC analysis and helped draft the manuscript; MAB performed the PMCA experiments. DLR performed the histological and neuropathological analysis; JWI performed neuropathological analysis, oversaw the study and reviewed and edited the manuscript. MWH conceived of the study, and participated in its design and coordination and helped to draft the manuscript. All authors read and approved the final manuscript.

## Additional file

## Electronic supplementary material

Additional file 1: Figure S1.: Western blot analysis of VPSPr and sCJD VV2 using anti-PrP primary antibodies recognising internal and C-terminal epitopes. Western blot analysis of cerebral cortex homogenate from an sCJD VV2 case or VPSPr case 1, with (+) or without (-) PK treatment, using the anti-PrP monoclonal antibodies 3F4 (amino acids 106-112) or 94B4 (C-terminal, amino acids 187-193). The volumes of homogenates analysed are indicated. Numbers in brackets [] indicate the volumes that were centrifugally concentrated. **Figure S2.** D-N and N values for whole brain homogenate versus centrifugal pellet for VPSPr versus non-CJD. D-N (red bar) values for brain frontal cortex homogenate and centrifugal pellet from a case of VPSPr and a non-CJD case (ALS/FTLD). Samples (100ml) of a 10% (w/v) homogenate of frontal cortex (H) from VPSPr case 1 or a non-CJD case were analysed directly by CDI. Alternatively, 100μl samples were centrifuged at 20,000g for 1h at 4°C and the pellets were analysed by CDI. The results are the means + S.D. for triplicate analyses of single samples. **Figure S3.** Equilibrium unfolding analysis. The stability of PrP^Sc^ in various regions of a VPSPr brain, two cases of GSS and a case of sCJD VV2 was investigated by CDI following denaturation with various concentrations of GdnHCl. The y-axis on this curve is the fraction of PrP^Sc^ that is unfolded, calculated as described in the methods. The abbreviations used for the various brain regions are the same as in Figure 5. For the analysis of FC, CB and PC, the data were merged from the analysis of 4, 3 and 2 samples, respectively. Single samples were analysed for the other regions. FC was analysed from one GSS case (P102L, small fragment), and Cb and FC was analysed from another GSS case (P102L, type 1). Underneath each figure is given the [GdnHCl]1/2 value and the 95% CI for the curve fit. (PDF 699 KB)

Below are the links to the authors’ original submitted files for images.

Authors’ original file for figure 1

Authors’ original file for figure 2

Authors’ original file for figure 3

Authors’ original file for figure 4

Authors’ original file for figure 5

Authors’ original file for figure 6

Authors’ original file for figure 7

Authors’ original file for figure 8

Authors’ original file for figure 9
