# Supplementary material for: The prion protein protease sensitivity, stability and seeding activity in variably protease sensitive prionopathy brain tissue suggests molecular overlaps with sporadic Creutzfeldt-Jakob disease
Source: Acta Neuropathol Commun. 2014 Oct 21;2:152. doi: 10.1186/s40478-014-0152-4 (PMC4210614; doi:10.1186/s40478-014-0152-4)
Supplement: Supplementary file 1 — Additional file 1: Figure S1.: Western blot analysis of VPSPr and sCJD VV2 using anti-PrP primary antibodies recognising internal and C-terminal epitopes. Western blot analysis of cerebral cortex homogenate from an sCJD VV2 case or VPSPr case 1, with (+) or without (-) PK treatment, using the anti-PrP monoclonal antibodies 3F4 (amino acids 106-112) or 94B4 (C-terminal, amino acids 187-193). The volumes of homogenates analysed are indicated. Numbers in brackets [] indicate the volumes that were centrifugally concentrated. Figure S2. D-N and N values for whole brain homogenate versus centrifugal pellet for VPSPr versus non-CJD. D-N (red bar) values for brain frontal cortex homogenate and centrifugal pellet from a case of VPSPr and a non-CJD case (ALS/FTLD). Samples (100ml) of a 10% (w/v) homogenate of frontal cortex (H) from VPSPr case 1 or a non-CJD case were analysed directly by CDI. Alternatively, 100μl samples were centrifuged at 20,000g for 1h at 4°C and the pellets were analysed by CDI. The results are the means + S.D. for triplicate analyses of single samples. Figure S3. Equilibrium unfolding analysis. The stability of PrPSc in various regions of a VPSPr brain, two cases of GSS and a case of sCJD VV2 was investigated by CDI following denaturation with various concentrations of GdnHCl. The y-axis on this curve is the fraction of PrPSc that is unfolded, calculated as described in the methods. The abbreviations used for the various brain regions are the same as in Figure 5. For the analysis of FC, CB and PC, the data were merged from the analysis of 4, 3 and 2 samples, respectively. Single samples were analysed for the other regions. FC was analysed from one GSS case (P102L, small fragment), and Cb and FC was analysed from another GSS case (P102L, type 1). Underneath each figure is given the [GdnHCl]1/2 value and the 95% CI for the curve fit. (PDF 699 KB) [file 40478_2014_9152_MOESM1_ESM.pdf]

Suppl. Fig 1

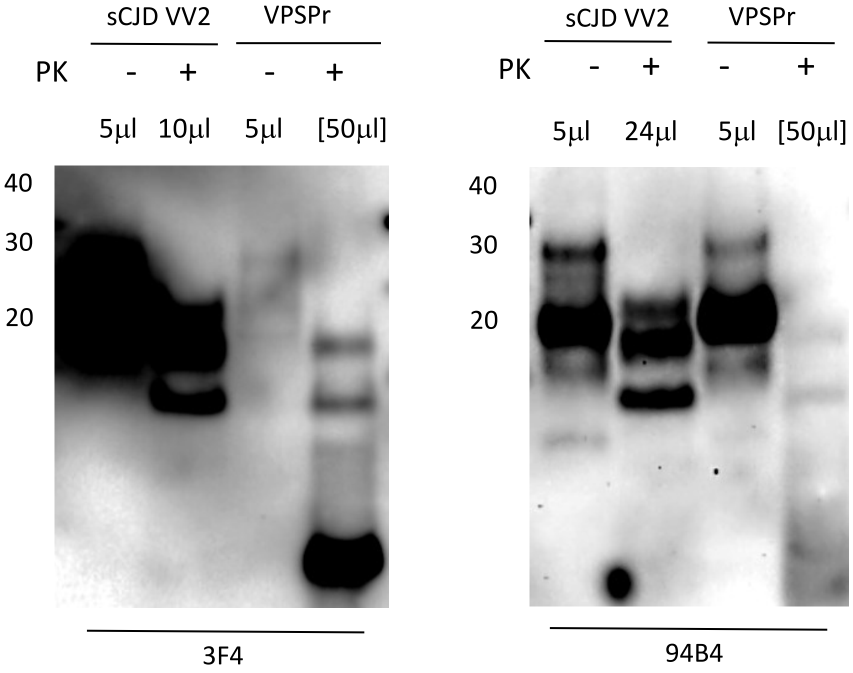

Suppl. Fig 1 Western blot analysis of VPSPr and sCJD VV2 using anti-PrP primary antibodies recognising internal and C-terminal epitopes.

Western blot analysis of cerebral cortex homogenate from an sCJD VV2 case or VPSPr case 1, with (+) or without (-) PK treatment, using the anti-PrP monoclonal antibodies 3F4 (amino acids 106-112) or 94B4 (C-terminal, amino acids 187-193). The volumes of homogenates analysed are indicated. Numbers in brackets [] indicate the volumes that were centrifugally concentrated.

Suppl. Fig 2

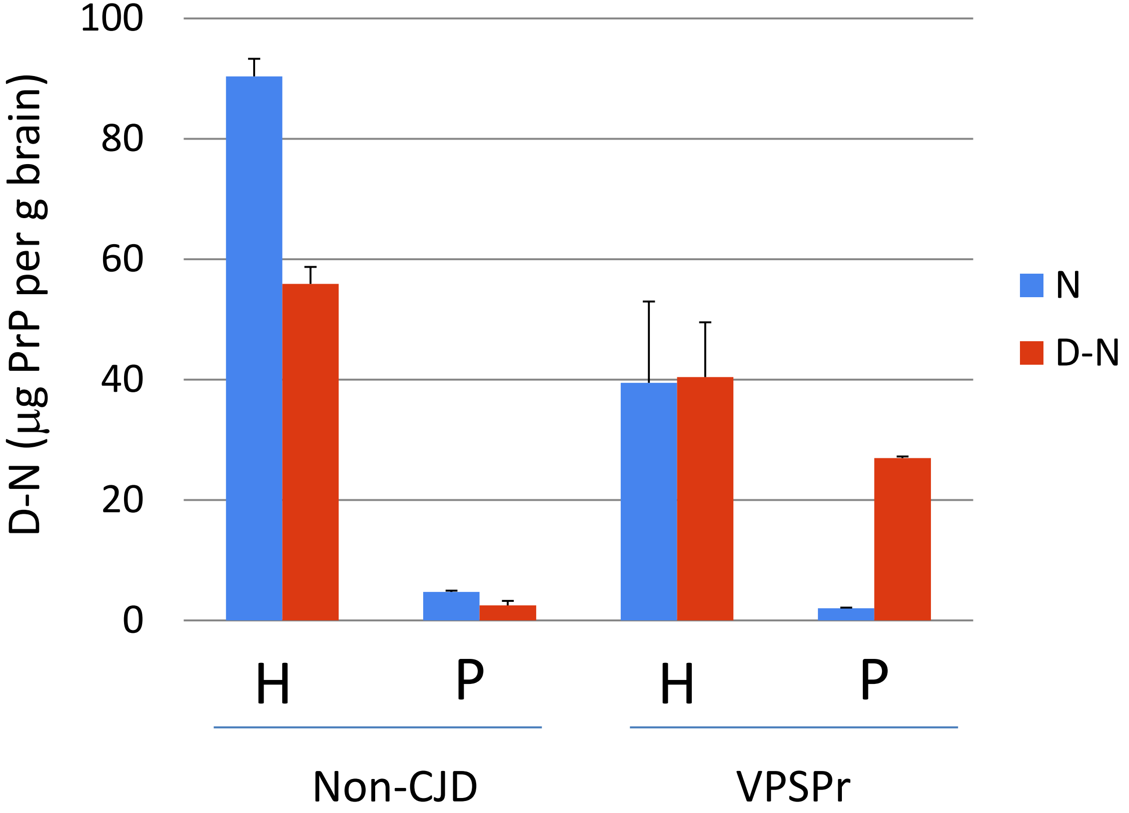

Suppl. Fig. 2 D-N and N values for whole brain homogenate versus centrifugal pellet for VPSPr versus non-CJD

D-N (red bar) values for brain frontal cortex homogenate and centrifugal pellet from a case of VPSPr and a non-CJD case (ALS/FTLD). Samples (100µl) of a 10% (w/v) homogenate of frontal cortex (H) from VPSPr case 1 or a non-CJD case were analysed directly by CDI. Alternatively, 100µl samples were centrifuged at 20,000g for 1h at 4°C and the pellets were analysed by CDI. The results are the means + S.D. for triplicate analyses of single samples.

Suppl. Fig. 3

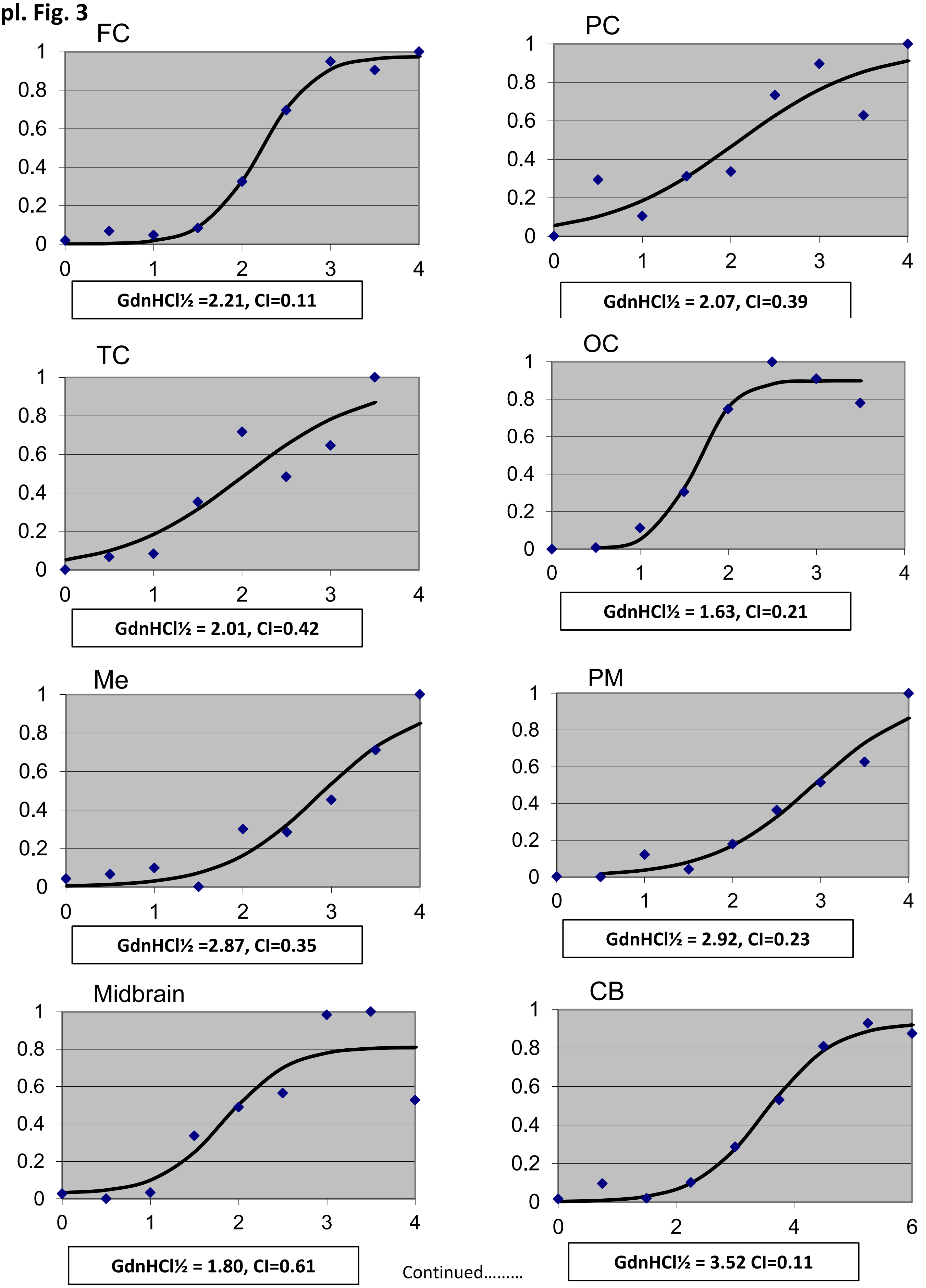

Continued………

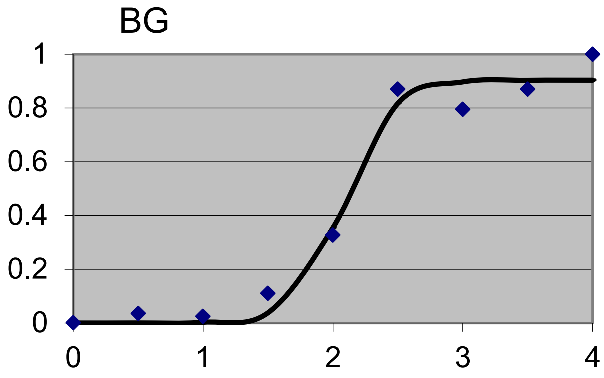

GdnHCl½ = 2.08, CI=0.21

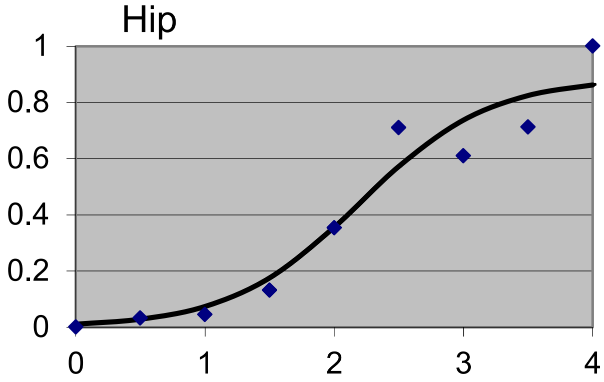

GdnHCl½ = 2.2, CI=0.26

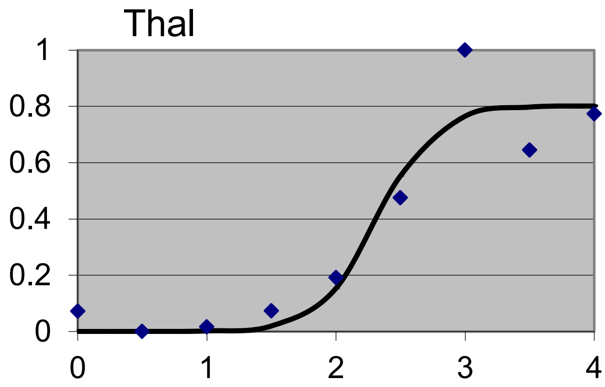

GdnHCl½ = 2.32, CI=0.31

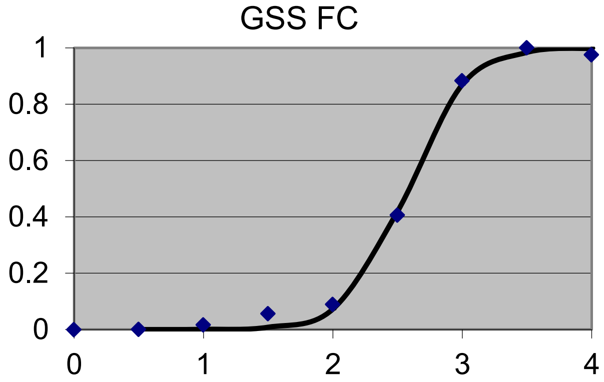

GdnHCl½ = 2.57, CI=0.06

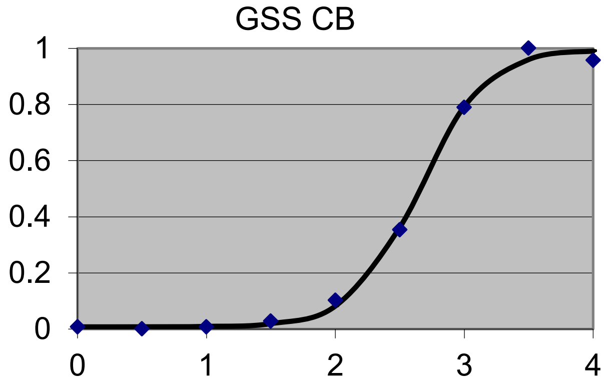

GdnHCl½ = 2.64, CI=0.06

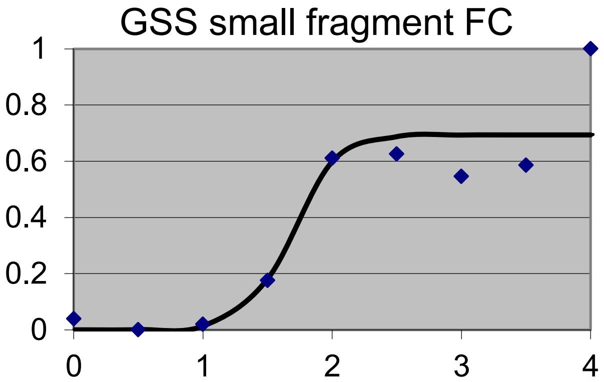

GdnHCl½ =1.68, CI=0.36

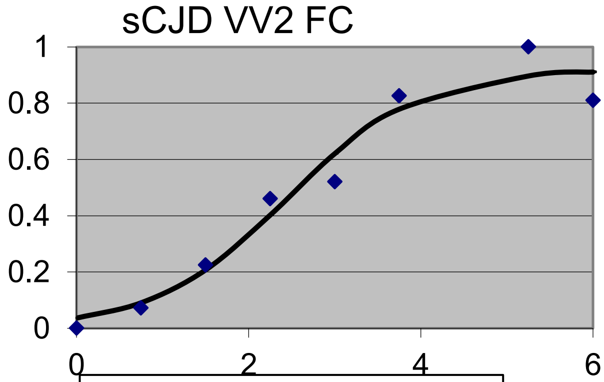

GdnHCl½ = 2.44, CI=0.22

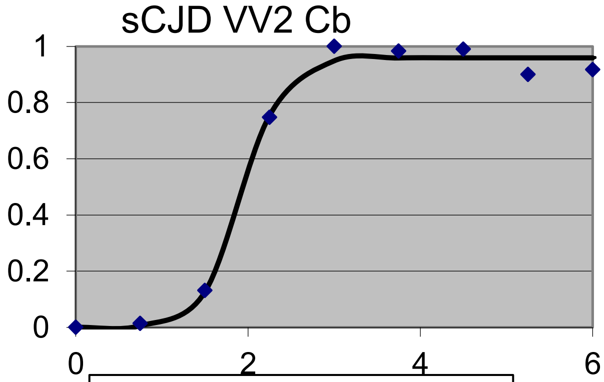

GdnHCl½ = 1.94, CI=0.10

Continued.........

Suppl. Fig. 3 Equilibrium unfolding analysis

The stability of PrP^{Sc} in various regions of a VPSPr brain, two cases of GSS and a case of sCJD VV2 was investigated by CDI following denaturation with various concentrations of GdnHCl. The y-axis on this curve is the fraction of PrP^{Sc} that is unfolded, calculated as described in the methods. The abbreviations used for the various brain regions are the same as in Fig. 5. For the analysis of FC, CB and PC, the data were merged from the analysis of 4, 3 and 2 samples, respectively. Single samples were analysed for the other regions. FC was analysed from one GSS case (P102L, small fragment), and Cb and FC was analysed from another GSS case (P102L, type 1). Underneath each figure is given the [GdnHCl]1/2 value and the 95% CI for the curve fit.
