# Supplementary figures and images for: The prion protein protease sensitivity, stability and seeding activity in variably protease sensitive prionopathy brain tissue suggests molecular overlaps with sporadic Creutzfeldt-Jakob disease
Source: Acta Neuropathol Commun. 2014 Oct 21;2:152. doi: 10.1186/s40478-014-0152-4 (PMC4210614; doi:10.1186/s40478-014-0152-4)

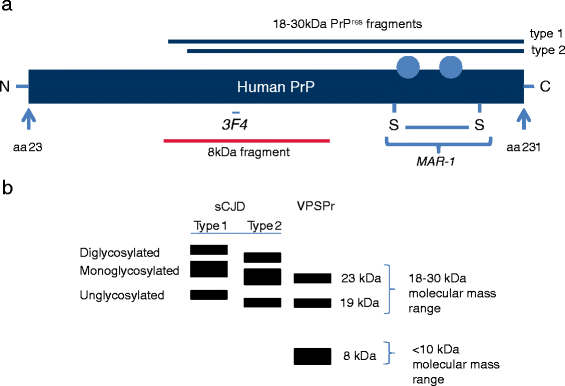

Supplement: Supplementary file 2 — Authors’ original file for figure 1 [file 40478_2014_9152_MOESM2_ESM.gif]

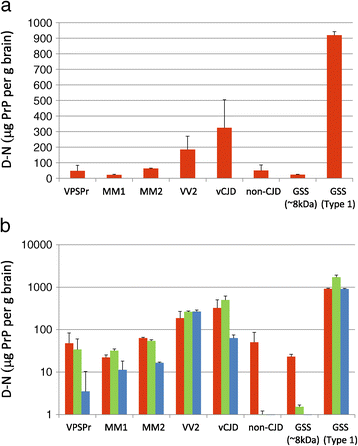

Supplement: Supplementary file 3 — Authors’ original file for figure 2 [file 40478_2014_9152_MOESM3_ESM.gif]

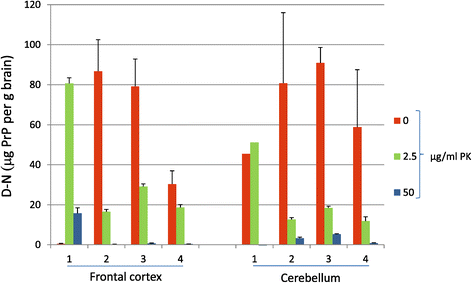

Supplement: Supplementary file 4 — Authors’ original file for figure 3 [file 40478_2014_9152_MOESM4_ESM.gif]

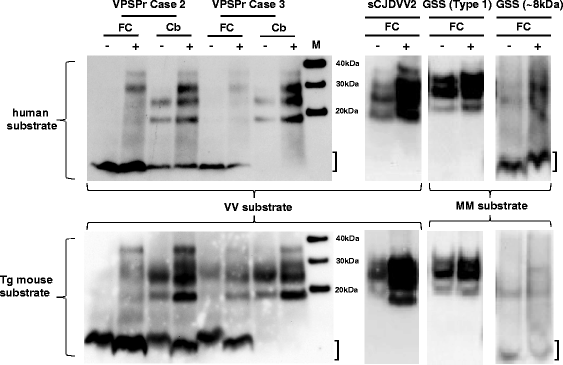

Supplement: Supplementary file 5 — Authors’ original file for figure 4 [file 40478_2014_9152_MOESM5_ESM.gif]

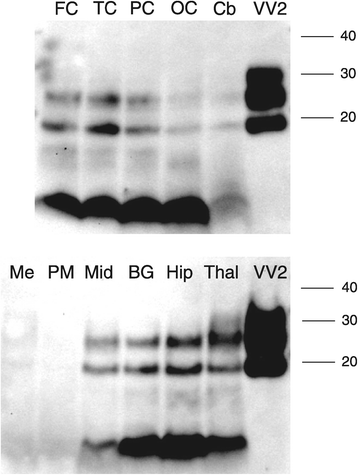

Supplement: Supplementary file 6 — Authors’ original file for figure 5 [file 40478_2014_9152_MOESM6_ESM.gif]

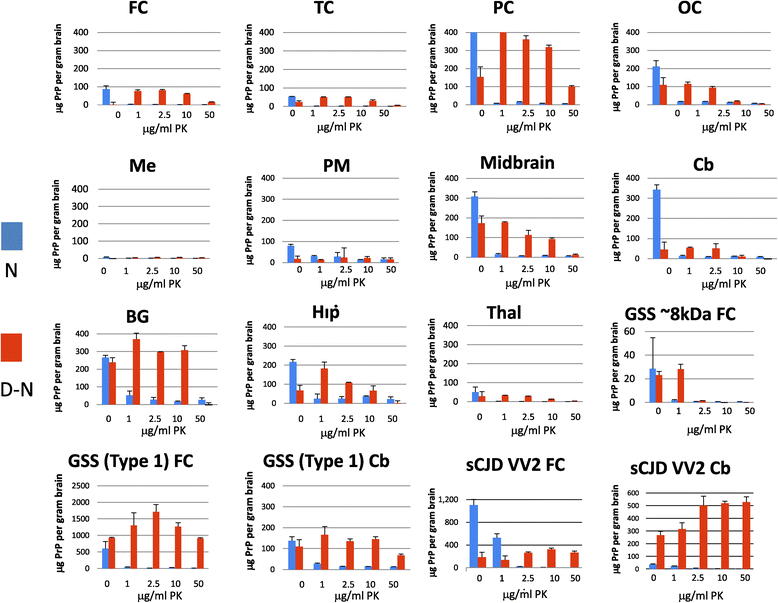

Supplement: Supplementary file 7 — Authors’ original file for figure 6 [file 40478_2014_9152_MOESM7_ESM.gif]

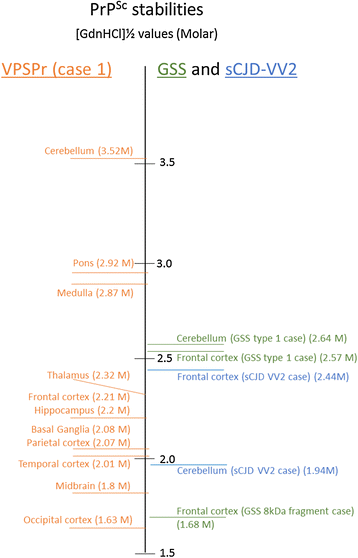

Supplement: Supplementary file 8 — Authors’ original file for figure 7 [file 40478_2014_9152_MOESM8_ESM.gif]

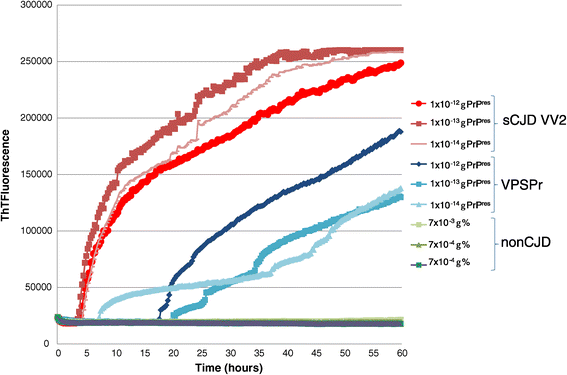

Supplement: Supplementary file 9 — Authors’ original file for figure 8 [file 40478_2014_9152_MOESM9_ESM.gif]

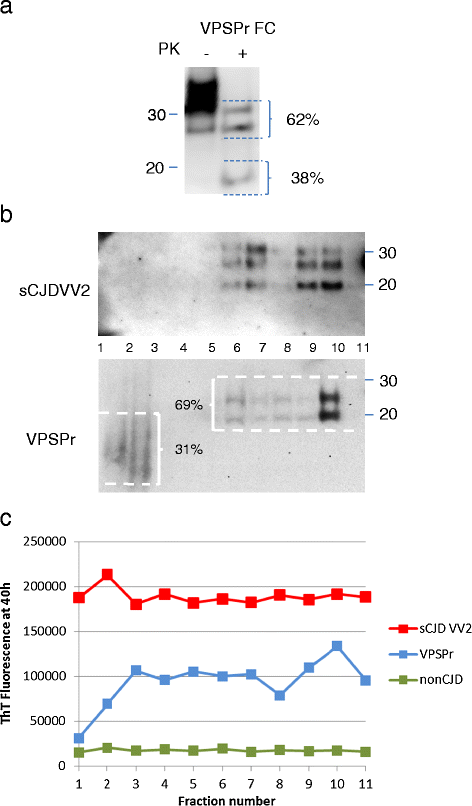

Supplement: Supplementary file 10 — Authors’ original file for figure 9 [file 40478_2014_9152_MOESM10_ESM.gif]
